# The Prevalence of Vitamin D Deficiency Is Similar between Thyroid Nodule and Thyroid Cancer Patients

**DOI:** 10.1155/2010/805716

**Published:** 2009-09-06

**Authors:** Nathan Laney, Jane Meza, Elizabeth Lyden, Judi Erickson, Kelly Treude, Whitney Goldner

**Affiliations:** ^1^Department of Internal Medicine, Section of Diabetes, Endocrinology, and Metabolism, University of Nebraska Medical Center, Omaha, NE 68198-3020, USA; ^2^Department of Biostatistics, College of Public Health, University of Nebraska Medical Center, Omaha, NE 68198-4375, USA

## Abstract

*Introduction*. There are reported associations between vitamin D deficiency and breast, prostate, and colon cancer, but the relationship in thyroid cancer has not been evaluated. *Methods*. We evaluated serum calcium, creatinine, albumin, and 25-hydroxy vitamin D (25-OH-D) in 42 thyroid nodule, 45 thyroid cancer in remission, and 24 active thyroid cancer patients. *Results*. 25-OH-D was not different between groups. The percent with 25-OH-D levels <75 nmol/L was not significantly different between groups and was not affected by season of measurement, age, or cancer stage. Multivariate regression showed a BMI of ≥30 kg/m^2^ to be the only significant predictor of vitamin D deficiency. *Conclusions*. Rates of vitamin D deficiency are similar in thyroid nodules and thyroid cancer, although higher than the general population. This is different than previous studies for other cancers, which show higher rates of vitamin D deficiency. BMI was the only predictor of vitamin D deficiency.

## 1. Introduction

Thyroid cancer is the most common endocrine-related malignancy. Thyroid cancer is more common in females, and is currently the seventh leading cause of new cancer diagnosis in women [1]. These cancers can range from papillary or follicular thyroid carcinoma, both well-differentiated tumors with excellent prognosis and indolent course, to poorly differentiated, and often lethal, anaplastic form [2]. Currently, known risk factors for thyroid cancer include previous high-dose environmental radioactive iodine exposure, previous childhood radiation to the head and neck, iodine deficiency, female gender, and family history of thyroid cancer [3, 4]. In 2007, there were estimated 33 550 new cases of thyroid cancer diagnosed, with estimated 1530 total deaths [5]. The incidence of new thyroid cancer diagnosis has increased by 6.2% from 1997 to 2004, with a concomitant increase in mortality by 0.3% from 1985 to 2004 [1]. It is unknown whether this rise in thyroid cancer is due to increased imaging of the neck resulting in increased diagnosis of smaller, nonpalpable cancers (mainly papillary thyroid carcinoma) that would have previously gone undiagnosed [6] or whether this is due to a true rise in thyroid cancer from an undetermined etiology.

Recently, studies have shown associations between vitamin D deficiency and breast [7], prostate [8], and colon [9] cancers. However, the association between vitamin D levels and thyroid cancer is unknown. There are animal models and in vitro studies that have shown vitamin D to have an antiproliferative effect on thyroid cancer [10–12].

This study evaluates vitamin D deficiency as not only a potential novel risk factor for thyroid cancer but also a potential therapeutic target. If thyroid cancer is similar to other solid tumors regarding its relationship to vitamin D, we hypothesize that vitamin D levels will be lower in patients with active thyroid cancer than patients with thyroid cancer in remission, and that both thyroid cancer groups will be lower than thyroid nodules.

## 2. Materials and Methods

### 2.1. Patients

This study was approved by the University of Nebraska Medical Center IRB to use stored blood specimens from the Thyroid Cancer Collaborative Registry (TCCR). The TCCR was established at UNMC/The Nebraska Medical Center in March 2008 and has accrued over 300 patients to date. The TCCR is a thyroid cancer and nodule database registry and biospecimen bank. All patients who give informed consent to be a part of the registry complete a standardized questionnaire that contains demographic information, medical history, family history, diet and lifestyle habits, past or current environmental exposures, and occupation. Blood and tissue samples are collected and stored for all patients with thyroid cancer and thyroid nodules.

This study is a pilot study designed to provide preliminary data, which will allow us to design a larger study. To design a larger trial, using the outcome “the proportion of persons with vitamin D deficiency” (defined as a baseline vitamin D level of <75 nmol/L), a sample size of 160 subjects (32 patients with residual disease, 64 patients in remission, and 64 noncancer controls with thyroid nodules) would achieve an 80% power to detect an effect size of 0.25 using 2 degrees of freedom Chi-Square Test with a significance level of 0.05. An effect size of 0.25 corresponds to vitamin D deficiency in approximately 32% of controls subjects, 59% of subjects in remission, and 59% of subjects with residual disease. Since this is a pilot study, we aimed to accrue 37% of the sample size of a fully powered study.

We evaluated 24 patients with active thyroid cancer, 45 patients with thyroid cancer in remission, and 42 patients with thyroid nodules. Patients were eligible if they had a history of thyroid cancer (papillary, follicular, follicular variant of papillary, or hurthle cell thyroid cancer), had a thyroid nodule, and were older than 19 years of age. At the time of the study, there were no cases of medullary or anaplastic thyroid cancers in the TCCR. Patients were also excluded if pregnant, or breast-feeding. Patients with a history of thyroid cancer were considered to be in remission if they met one of the following criteria: (1) total thyroidectomy and radioactive iodine remnant ablation and unstimulated thyroglobulin <1 ng/mL and/or stimulated thyroglobulin <5 ng/mL and >6 months since initial treatment or >12 months since repeat treatment; (2) total thyroidectomy and no radioactive iodine remnant ablation and negative neck ultrasound; (3) less than total thyroidectomy and no radioactive iodine remnant ablation and negative neck ultrasound; (4) detectable antithyroglobulin antibodies and negative imaging (radioactive iodine whole body scan or ultrasound). Patients with a history of thyroid cancer were considered to have active disease if they met one of the following criteria: (1) total thyroidectomy and radioactive iodine remnant ablation and unstimulated thyroglobulin >1 ng/mL and/or stimulated thyroglobulin >5 ng/mL; (2) Less than 6 months from initial treatment or <12 months from treatment for recurrent or persistent disease; (3) Evidence of gross recurrence by imaging. Thyroid nodules were included if they had thyroid nodules confirmed by ultrasound, regardless of size. Patients with thyroid nodules were on thyroid hormone replacement if clinically indicated, but all were not placed on thyroid hormone empirically. All thyroid cancer patients were on thyroid hormone replacement to suppress TSH as indicated by current thyroid cancer treatment standards.

### 2.2. Methods

Demographic information including age, gender, BMI, season of vitamin D measurement was obtained in addition to histologic type of thyroid cancer, TNM staging, and vitamin D supplementation history. The chart was also reviewed for baseline vitamin D measurement in patients who were taking >800 IU vitamin D per day. Stored serum from the TCCR was assessed for serum calcium, albumin, creatinine, and 25-hydoxy-vitamin D (25-OH-D). Serum utilized to run laboratories was obtained from participants over a period starting 4/1/2008 and ending on 2/6/2009. The serum calcium, albumin, and creatinine were evaluated at the UNMC/NMC laboratories utilizing Ortho Clinical Diagnostics Vitros 950 system. For those individuals taking supplemental vitamin D exceeding 800 IU per day, we utilized baseline 25-OH-D levels prior to vitamin D supplementation, if available. Baseline 25-OH-D levels were obtained from the electronic medical record, and extend from 12/15/2001 through 2/2/2009. However, 25-OH-D levels prior to 07/22/2008 were sent to ARUP for analysis that used either DiaSorin or Nichols assays during that period. All 25-OH-D levels starting 07/23/2008 were done through the UNMC/NMC laboratories, utilizing tandem mass spectrometry on the Applied Biosystems API3000 MS/MS equipped with Agilent 1100 LC. Baseline 25-OH-D levels prior to July 2008 (ARUP assays) were obtained from 11 thyroid nodules, 10 thyroid cancer in remission, and 2 active thyroid cancer patients. There were 2 thyroid nodules, 1 thyroid cancer in remission, and 4 active thyroid cancer patients who did not have baseline 25-OH-D levels available, or were on an unknown dose of vitamin D, and stored serum was used to evaluate 25-OH-D levels.

### 2.3. Analysis

Patient characteristics for each group were assessed using descriptive statistics, including medians, range, frequencies, and proportions. Categorical outcomes were compared between the 3 groups using a Fisher's exact test. Continuous outcomes were compared between the groups using the Kruskal Wallis test with subsequent pair-wise comparisons performed using a Wilcoxon rank sum test and a Bonferroni-adjusted alpha level of 0.017 when the overall test was significant. A logistic regression modeling procedure was used to compare the odds of low 25-OH-D levels (vitamin D <75 versus vitamin D ≥75 nmol/L) between patient groups while adjusting for other risk factors: gender, age (19–45 years versus >45 years), BMI (<30 versus >30 kg/m^2^), season of vitamin D measurement (winter: October–March; summer: April–September), and TNM staging. Only risk factors significant at the 0.10 level in univariate analysis were included in the multivariate model. A value of *P* < .05 was considered significant for all other comparisons.

Tandem mass spec for vitamin D assay was used for all patients that were not on supplemental vitamin D >800 IU per day. If they were on vitamin D >800 IU per day, baseline vitamin D levels, utilizing either Diaosrin or Nichols assays, were used in the analysis as described earlier. Because of the potential for differences in vitamin D levels obtained with different assays, we also analyzed our data using only the patients with vitamin D levels assessed by the tandem mass spec method, which included the majority of patients in the study (81%). We did have a small number of patients (13%) with samples run on both the Diasorin and mass spec assays available, so we also compared the mean ± SD of vitamin D of the 2 assays.

This research was funded by a grant from the University of Nebraska Medical Center Center for Clinical and Translational Research.

## 3. Results and Discussion

A total of 111 patients were evaluated, 42 (38%) nodules, 45 (40%) thyroid cancer in remission, and 24 (22%) active thyroid cancer. Baseline characteristics are provided in Table 1. Gender, age, season, and BMI were not significantly different between groups, however, there was a trend toward more males in the active thyroid cancer group. All patients enrolled in this study were Caucasian. Ninety percent of the nodule group was female compared with 87% in remission and 71% percent in the active thyroid cancer group (*P* = .11). Fifty-seven percent of the nodule group was >45 years, compared with 44% of the remission group and 37% of the active group (*P* = .28). Summer season of vitamin D measurement was 62% in the nodule group, 64% in the remission group, and 67% in the active group (*P* = .94). There was no significant difference between the remission and active thyroid cancer groups when evaluating for TNM stage (*P* = .20) or histologic type of thyroid cancer (*P* = .35). Calcium, albumin, and creatinine were not statistically different between groups (data not shown).

After removal of the Diasorin and Nichols assays, a total of 90 patients were evaluated. This included 33 (37%) nodules, 35 (39%) thyroid cancer in remission, and 22 (24%) active thyroid cancer patients. Using only the mass spec vitamin D samples, gender, age, season, and BMI were not significantly different. However, TNM stage was significantly different between the remission and active cancer groups showing a higher percentage of the remission group was stage I. In the remission group, 82% were stage I, 9% were stage II, 3% were stage III, and 6% were stage IVa, while in the active cancer group, 55% were stage I, 15% were stage II, 22% were stage III, and 5% were stage IVa (*P* = .05). Histologic type of cancer was not significantly different.

Vitamin D deficiency was defined as 25-OH-D <75 nmol/L. When evaluating each group (using all vitamin D assays) for percentage of patients with vitamin D deficiency, there was no significant difference (Table 2). Fifty-two percent of thyroid nodules, 44% of thyroid cancer in remission, and 42% of active thyroid cancer patients were vitamin D deficient (*P* = .67). When stratifying each group by vitamin D tertiles (<25 nmol/L versus 25–75 nmol/L versus >75 nmol/L), there again was no significant difference (*P* = .74) (Table 2). In the nodule versus remission versus active groups, 2%, 4%, and 0% were <25 nmol/L, respectively. However, 52%, 40%, and 46% had 25-OH-D levels between 25–75 nmol/L, respectively, and 45%, 56%, and 54% had 25-OH-D levels of >75 nmol/L, respectively. After removing the Diasorin and Nichols assays, there was still no significant difference when evaluating each group for percentage of patients with vitamin D deficiency or when stratifying each group by tertiles (Table 2).

When evaluating median (range) 25-OH-D for the entire group (using all vitamin D assays), the summer group (*n* = 71) had a median 25-OH-D of 73 (<25–180) nmol/L, and the winter group (*n* = 40) had a median of 93 (<25–218) nmol/L (*P* = .56). In the nodule group, the summer group (*n* = 26) had a median of 76.3 (33–165) nmol/L and the winter group (*n* = 16) had a median of 70 (<25–128) nmol/L (*P* = .13). In the remission group, the summer group (*n* = 29) had a median of 65 (<25–180) nmol/L and the winter group (*n* = 16) had a median of 95 (38–148) nmol/L (*P* = .14). In the active group, the summer group (*n* = 16) had a median of 80 (30–128) nmol/L and the winter group (*n* = 8) had a median of 108 (45–218) nmol/L (*P* = .20). When evaluating the distribution in tertiles, no significant difference was observed (data not shown).

After removal of vitamin D levels obtained from Diasorin and Nichols assays, there was no significant difference when evaluating the median (range) 25-OH-D of the entire group, or any of the groups individually. In the group as a whole, the summer group (*n* = 63) had a median 25-OH-D of 80 (<25–180) nmol/L, and the winter group (*n* = 40) had a median of 93 (25–218) nmol/L (*P* = .28). In the nodule group, the summer group (*n* = 24) had a median of 88 (33–165) nmol/L and the winter group (*n* = 9) had a median of 73 (25–128) nmol/L (*P* = .18). In the remission group, the summer group (*n* = 24) had a median of 68.8 (<25–180) nmol/L and the winter group (*n* = 11) had a median of 98 (38–148) nmol/L (*P* = .10). In the active group, the summer group (*n* = 15) had a median of 85 (30–128) nmol/L and the winter group (*n* = 7) had a median of 120 (48–218) nmol/L (*P* = .10). When evaluating the distribution in tertiles, no significant difference was observed (data not shown).

Cancer stage and vitamin D levels were not statistically different (Table 3). In the remission group, stage I had a median (range) 25-OH-D of 84 (<25–180) nmol/L, stage II had a median of 60 (45–158) nmol/L, stage III had a median of 93 (38–135) nmol/L, and stage IVa had a median of 93 (65–118) nmol/L (*P* = .92). In the active group, stage I had a median 25-OH-D of 84 (30–218) nmol/L, stage II had a median of 95 (85–108) nmol/L, stage III had a median of 80 (48–88) nmol/L, and stage IVa had a median of 95 (45–95) nmol/L (*P* = .71). After removing the vitamin D levels obtained from Diasorin and Nichols assays, no statistical difference was observed when evaluating cancer stage and vitamin D levels (Table 3).

Vitamin D levels in different histologic types of thyroid cancer were also not statistically different (Table 4), however there was a trend for papillary thyroid cancer to have higher median levels of vitamin D compared with other histologic types of thyroid cancer (using all vitamin D assays). Median (range) 25-OH-D for papillary thyroid cancer was 89 (<25–218) nmol/L and the other histologic types combined were 60 (38–180) nmol/L (*P* = .28). The same findings were observed when removing vitamin D levels obtained from Diasorin and Nichols assays from our analysis (Table 4).

However, BMI >30 kg/m^2^ was the only significant risk factor for vitamin D deficiency in univariate analysis (OR 6.98, 95% CI:2.95–16.52, *P* < .0001). This held true when analyzing the data using only tandem mass spec vitamin D levels (OR 9.33, 95% CI:3.50–24.88, *P* < .0001). Multivariate analysis after adjustment for BMI revealed that cancer status was not a significant predictor for vitamin D deficiency compared to the nodule group. BMI >30 kg/m^2^, however, remained a statistically significant risk factor for vitamin D deficiency (OR 7.13, 95% CI:2.99–17.01, *P* < .0001). As BMI was the only significant risk factor for vitamin D deficiency, we compared the group as a whole, and individually. When using all vitamin D specimens, BMI ≥30 kg/m^2^ was significantly associated with vitamin D deficiency in the entire group (*P* < .0001), thyroid nodule group (*P* = .0001), and active thyroid cancer group (*P* = .04), but not the thyroid cancer in remission group (*P* = .12). When using only tandem mass spec vitamin D levels, BMI ≥30 kg/m^2^ was significantly associated with vitamin D deficiency in the entire group (*P* < .0001), thyroid nodule group (*P* = .0004), and thyroid cancer in remission group (*P* = .03), but not the active thyroid cancer group (*P* = .08).

We had 14 patients who had a clinical sample run using the Diasorin assay and a stored research sample run later using mass spec from the same day. We found the mean ± SD for the mass spec assay of vitamin D level was 94.7 ± 35.8 nmol/L, while the Diasorin assay vitamin D level was 84 ± 43.1 nmol/L. The mean difference between the two assays was 10.5 ± 22 nmol/L (95% CI: −2.13–23.18), showing no significant difference between the assays.

## 4. Conclusion

This pilot study shows levels of vitamin D and rates of vitamin D deficiency to be similar between patients with thyroid nodules, thyroid cancer in remission, and active thyroid cancer. We did not find an association between vitamin D deficiency and the histologic type of thyroid cancer, the stage of thyroid cancer, or the status of the disease. The only risk factor found to be independently associated with vitamin D deficiency was BMI >30 kg/m^2^, consistent with previous studies [13, 14].

These results are different from other studies that have shown a relationship between vitamin D deficiency and other solid tumors. One recent study evaluated the incident risk of developing nonskin cancers of all types when vitamin D supplementation was administered over four years [15]. This study compared individuals receiving calcium plus 1000 IU of vitamin D supplementation to calcium supplementation only and placebo controls. All individuals had similar baseline mean 25-OH-D levels around 70 nmol/L, with a mean increase in 25-OH-D levels of 24 nmol/L at 12 months in the group receiving vitamin D only. In comparison to the placebo group, the calcium plus vitamin D group had a lower relative risk of developing cancer over the course of the study. Epidemiological studies have also shown an increased incidence of colon cancer with lower levels of vitamin D. Vitamin D levels in the lowest quartile of vitamin D, or below 75 nmol/L, have been associated with approximately twice the risk of colon cancer than those with higher levels [9, 16]. Levels of 25-OH-D in postmenopausal women with breast cancer have been shown to be lower than women without breast cancer and there is a significant inverse association between 25-OH-D levels and postmenopausal breast cancer [7]. Another study evaluating prostate cancer and vitamin D also demonstrated risk was inversely related to 25-OH-D, but the risk in this study was only seen at levels <40 nmol/L [8]. Using thyroid nodules as noncancer controls, we did not see a significant difference between the thyroid cancer groups, active or remission, or the thyroid nodules. This needs to be investigated further. This could be due to thyroid nodules not truly being “healthy” controls, and may represent early thyroid pathology, or early thyroid cancers. It could also be that thyroid cancer generally has a less aggressive course as compared with breast, colon, and some forms of prostate cancer and may not be associated with vitamin D status.

The only risk factor for vitamin D deficiency in our study was BMI, consistent with previous studies [13, 14]. Season of vitamin D measurement has previously been shown to affect vitamin D levels, especially in northern latitudes where there is no cutaneous conversion of vitamin D in the skin during the winter months [17, 18]. Our study, however, did not see a marked difference in the groups in summer versus winter. One explanation could be that we did not have a summer and winter sample from each person. Another explanation could be that vitamin D levels may be less dependent on cutaneous conversion of vitamin D when associated with thyroid nodules or cancer. Interestingly, the median levels, when comparing all groups together, were higher during the winter months. However, when categorized into tertiles, the 25–75 and >75 nmol/L range had a higher percentage of individuals from the summer group. Although it is possible that having more summer specimens could affect these results, post-hoc analysis did not show a statistically significant effect of season on vitamin D levels.

Previous reports of vitamin D deficiency in healthy populations have been roughly 14–34% [14, 18, 19]. We overall found higher rates of vitamin D deficiency in all three of our groups. The prevalence of vitamin D deficiency in nodules was 48%, thyroid cancer in remission was 56%, and active thyroid cancer was 58%. Even though the rates were not statistically different between groups, all three groups were higher than what has been previously reported in healthy subjects. Ethnicity has been associated with vitamin D levels [20, 21], so it is important to consider when comparing data. Our study participants were predominantly Caucasians, so should be compared to normal controls with similar ethnicity. Recently, NHANES data showed there has been an overall decline in 25-OH-D levels from the 1988–1994 census compared with the 2001–2004 census [19]. This could explain the increased rates of overall vitamin D deficiency in all three of our groups, however, there are many potential reasons for this change, one of which includes changes and overall improvement in the vitamin D assay. Other potential causes could be difference in ethnicity, season of measurement of vitamin D, and BMI of the population as a whole. All of these issues need to be considered when comparing the data.

One of the main limitations of this study is the lack of healthy (no thyroid disease) controls. Unfortunately, healthy patients without thyroid disease are not part of the TCCR database. However, in a separate, published study [14], healthy controls were recruited and vitamin D levels obtained at our institution, and we use them as a basis for comparison since they are demographically similar to our current study group. In that study, there were 41 healthy control, nonobese participants with a mean age was 46.3 years. However, Seventy-eight percent were female, 98% were Caucasian, and they had a mean BMI of 24.7 kg/m^2^. That group is also representative of our study group because it represents patients from the same latitude, and included vitamin D levels from both summer (54%) and winter (46%) months. The prevalence of vitamin D deficiency in that healthy control population was 32% [14] as compared with our data showing 48% of thyroid nodules with vitamin D deficiency and 56–58% of thyroid cancer.

Another limitation is the small sample size. As this is a pilot study, our numbers are small, which may have prevented us from detecting a statistical difference. Our results, therefore, will need to be confirmed with a larger, potentially multicenter study. It is important to consider that some autoimmune conditions have been reported to be associated with vitamin D deficiency, which may play a role in our thyroid nodule group. Thyroid antibodies are not routinely obtained for all thyroid nodule patients, so were not available from all patients. However, when evaluating those patients that did have antibodies available, there was only 14% of the entire group that had positive antibodies.

We recognize that using different vitamin D assays may result in different vitamin D levels reported. To account for this potential difference, we have analyzed our data using results combined from the three different assays, as well as tandem mass spec alone, which make up the majority of specimens (81%). We did not find any significant differences in our results when analyzing data from the whole group and mass spec assay only, and there was no significant difference between vitamin D levels when comparing simultaneous Diaosrin and mass spec results.

In conclusion, we did not find a significant association between vitamin D levels and thyroid nodules, thyroid cancer in remission, and active thyroid cancer. This is different than studies looking at other solid tumors and warrants further investigation. We also found the overall prevalence of vitamin D deficiency to be higher than expected for the general healthy population, which also needs to be studied further in a larger population that controls for issues known to affect vitamin D such as ethnicity, BMI, season of measurement, autoimmunity, and vitamin D supplementation.

## Figures and Tables

**Table 1:** Baseline Characteristics.

Characteristic	Nodule (*n* = 42)	Remission (*n* = 45)	Active (*n* = 24)	
Sex	Count (%)	Count (%)	Count (%)	*P*-value
* *Female	38 (90)	39 (87)	17 (71)	
* *Male	4 (10)	6 (13)	7 (29)	.11

Age group				
* *Older (>45years)	24 (57)	20 (44)	9 (37)	
* *Younger (19–45 years)	18 (43)	25 (56)	15 (63)	.28

Season				
* *Summer	26 (62)	29 (64)	16 (67)	
* *Winter	16 (38)	16 (36)	8 (33)	.94

TNM stage				
* *1	na	31 (74)	12 (55)	
* *2	na	5 (12)	3 (14)	
* *3	na	3 (7)	5 (23)	
* *4A	na	3 (7)	1 (5)	
* *4B	na	0	1 (5)	.20*

CA pathology				
* *Nodule	42 (100)	0	0	
* *Papillary	0	34 (76)	22 (92)	
* *Follicular	0	4 (9)	1 (4)	
* *Follicular variant of papillary	0	7 (16)	1 (4)	.35*

Race				
* *Caucasian	42 (100)	45 (100)	24 (100)	

BMI (kg/m^2)^	Median (range)	Median (range)	Median (range)	
	28.0 (19.8–51.7)	27.4 (17.8–43.0)	29.0 (16.7–46.0)	.66

*P-values for TNM stage and CA pathology were based on comparisons between the remission and active cancer groups only.

**Table 2:** Distribution of vitamin D levels for each group.

All vitamin D assays	Nodule (*n* = 42)	Remission (*n* = 45)	Active (*n* = 24)	
Vitamin D group	Count (%)	Count (%)	Count (%)	*P*- value
* *<25 nmol/L	1 (2)	2 (4)	0	
* *25–75 nmol/L	22 (52)	18 (40)	11 (46)	
* *>75 nmol/L	19 (45)	25 (56)	13 (54)	.74

Vitamin D group				
* *<75 nmol/L	22 (52)	20 (44)	10 (42)	
* *≥75 nmol/L	20 (48)	25 (56)	14 (58)	.67

Tandem mass spec assay	Nodule (*n* = 33)	Remission (*n* = 35)	Active (*n* = 22)	

Vitamin D group	Count (%)	Count (%)	Count (%)	*P*- value
* *<25 nmol/L	0	1 (3)	0	
* *25–75 nmol/L	17 (52)	14 (40)	9 (41)	
* *>75 nmol/L	16 (48)	20 (57)	13 (59)	.75

Vitamin D group				
* *<75 nmol/L	16 (48)	15 (43)	8 (36)	
* *≥75 nmol/L	17 (52)	20 (57)	14 (64)	.32

**Table 3:** Thyroid Cancer Stage and Median 25-OH-D levels.

TNM stage	*N*	Median nmol/L	Minimum nmol/L	Maximum nmol/L	*P*- value
All vitamin D assays					

Remission					
* *1	30	84	<25	180	
* *2	5	60	45	158	
* *3	3	93	38	135	
* *4A	3	93	65	118	.92

Active					
* *1	12	84	30	218	
* *2	3	95	85	108	
* *3	5	80	47	88	
* *4A	2	95	45	95	.71

Tandem mass spec vitamin D assays					

Remission					
* *1	27	88	<25	180	
* *2	3	58	45	158	
* *3	1	93	93	93	
* *4A	2	91.3	65	118	.93

Active					
* *1	11	95	30	218	
* *2	3	95	85	108	
* *3	5	75	48	88	
* *4A	1	95	95	95	.61

**Table 4:** Histologic Type of Thyroid Cancer and 25-OH-D levels.

Cancer pathology	*N*	Median nmol/L	Minimum nmol/L	Maximum nmol/L	*P*-value
All vitamin D assays					
* *Papillary	56	89	<25	218	
* *Other	13	60	38	180	0.28

Tandem mass spec Vitamin D assay					
* *Papillary	47	88	<25	218	
* *Other	10	73.8	43	180	0.61
